# Mucilage of *Coccinia grandis* as an Efficient Natural Polymer-Based Pharmaceutical Excipient †

**DOI:** 10.3390/polym14010215

**Published:** 2022-01-05

**Authors:** Kumbakonam Balachandran Ilango, Senguttuvan Gowthaman, Kumbakonam Ilango Seramaan, Kumarappan Chidambaram, Mohammad F. Bayan, Mohamed Rahamathulla, Chandrasekaran Balakumar

**Affiliations:** 1 Department of Pharmaceutics, College of Pharmacy, Shree Venkateshwara College of Paramedical Sciences, Erode 638455, India; gowthamanpharma@gmail.com; 2 Department of Pharmaceutics, Vellalar College of Pharmacy, Erode 638012, India; seramaan5435@gmail.com; 3 Department of Pharmacology, School of Pharmacy, King Khalid University, Abha 62529, Saudi Arabia; 4 Faculty of Pharmacy, Philadelphia University, P.O. Box 1, Amman 19392, Jordan; mbayan01@qub.ac.uk; 5 Department of Pharmaceutics, School of Pharmacy, King Khalid University, Abha 62529, Saudi Arabia; rahapharm@gmail.com; 6 Department of Pharmaceutical Chemistry, School of Pharmacy, ITM University, Gwalior 474001, India; dhillbalu@gmail.com

**Keywords:** binding agent, disintegrating agent, natural polymer, mucilage, *Coccinia grandis*

## Abstract

Natural eco-friendly materials are recently employed in products to replace synthetic materials due to their superior benefits in preserving the environment. The herb *Coccinia grandis* is widely distributed in continents like Asia and Africa and used traditionally to treat fever, leprosy, asthma, jaundice, and bronchitis. Mucilage of *Coccinia grandis* was accordingly extracted, isolated by a maceration technique, and precipitated. The mucilage was evaluated for its physicochemical, binding, and disintegrant properties in tablets using paracetamol as a model drug. The crucial physicochemical properties such as flow properties, solubility, swelling index, loss on drying, viscosity, pH, microbial load, cytotoxicity was evaluated and the compatibility was analyzed using sophisticated instrumental methods (TGA, DTA, DSC, and FTIR). The binding properties of the mucilage was used at three different concentrations and compared with starch and PVP as examples of standard binders. The disintegrant properties of mucilage were used at two different concentrations and compared with standard disintegrants MCCP, SSG, and CCS. The tablets were punched and evaluated for their hardness, friability, assay, disintegration time, in vitro dissolution profiles. In vitro cytotoxicity studies of the mucilage were performed in a human embryonic kidney (HEK) cell line. The outcome of the study indicated that the mucilage had good performance compared with starch and PVP. Further, the mucilage acts as a better disintegrant than MCCP, SSG and CCS for paracetamol tablets. Use of a concentration of 3% or less demonstrated the ability of the mucilage to act as a super disintegrating agent and showed faster disintegration and dissolution, which makes it as an attractive, promising disintegrant in formulating solid dosage forms to improve the therapeutic efficacy and patient compliance. Moreover, the in vitro cytotoxicity evaluation results demonstrated that the mucilage is non-cytotoxic to human cells and is safe.

## 1. Introduction

The oral route is the most preferred route of administration of various drugs as it is considered to be the convenient, safe, and painless [1]. Recently, in the novel drug delivery system (NDDS), various researchers developed convenient solid dosage forms and achieved better patient compliance [2]. Various plant gums and mucilages have been used as natural binders and disintegrants in the formulation of tablets [3]. However, the identification of an optimized, novel and effective binder and disintegrant for the manufacturing of tablets in the pharmaceutical industry remains a challenging task [4]. Binders and disintegrants are major pharmaceutical excipients that play an important role in solid dosage forms [5]. Binders are employed in tablet formulations to impart cohesion to powder mixtures and hence improve the flow properties of the granules. Thy are used to hold various powders intact together to form a tablet. The wet binder is the most important ingredient in the wet granulation process, and most binders are substances that are hydrophilic and soluble in water such as starch, gelatin, polyvinylpyrrolidone (PVP), etc. [6]. Disintegrating agents are substances routinely incorporated in tablet formulations to assist the breakup of the compacted mass when it is kept into a fluid environment. They also enhance the penetration of moisture and dispersion of the tablet matrix. Notably, the commonly used disintegrating agents in the tablet formulations are microcrystalline cellulose powder (MCCP), croscarmellose sodium (CCS), and sodium starch glycollate (SSG) [7].

*Coccinia grandis* (L.) Voigt, commonly known as ivy gourd, belongs to the pumpkin (Cucurbitaceae) family. It is frequently found in Asian countries such as India, Pakistan, and Sri Lanka and also distributed in Tropical Africa [8]. *Coccinia grandis* is a tropical aggressive climbing vine. It is an outdoor plant and can spread vegetatively or by seeds. It is oval with a green to red color. The skin is thick, sticky, and hairless. It becomes sweet, soft, and juicy once it is ripened. In the traditional system of medicine, fruits of *Coccinia grandis* have been used to treat leprosy, fever, asthma, bronchitis, and jaundice [9,10,11,12,13]. Recently, it was discovered that the polysaccharide found in *Coccinia grandis* can be used as an anti-diabetic agent [14]. Considering all the above literature reports, the current study was designed to incorporate the isolation of the mucilage from the fruits of *Coccinia grandis*, evaluation, and its potential applications as a novel binding and disintegrating agent in the tablet formulations.

Paracetamol, also known as acetaminophen or *N*-acetyl-*p*-aminophenol is widely used over-the-counter analgesic (pain reliever) and antipyretic (fever reducer) was selected in our study as a model drug [15]. Paracetamol is a drug with known capping and lamination problems that normally requires an appropriate binder for its formulation and upon storage, paracetamol tablets gain hardness, thus normally requiring a disintegrant to form ideal tablets. It was found in the literature that the mucilage of this fruit was not studied or reported for its binding and disintegrant properties. Hence, we are interested to investigate for the first time, the binding and disintegrant properties of the mucilage of *Coccinia grandis* in the tablet formulation and comparing its efficiency with the available standard binding and disintegrating agents.

## 2. Materials and Methods

### 2.1. Materials

Paracetamol (Granules India), Starch (Ridhi Siddhi, Ahmedabad, India), croscarmellose sodium (Shreeji Pharma, Gujarat, India), microcrystalline cellulose powder (Loba Chemie Pvt Ltd., Mumbai, India), sodium starch glycollate (J.R. Pharma, Gujarat, India), polyvinylpyrrolidone (J.B. Khokhani & Co., Mumbai, India), sodium methylparaben, sodium propylparaben (Nebula Health Care, Gujarat, country), magnesium stearate (Pantogan Chemicals, Chennai, India), talc (Supreme Traders, Coimbatore, India) were received as gift samples from Kreszent Pharma (Pondicherry, India). The fruits of *Coccinia grandis* were procured from a local market in Coimbatore, India and the same was identified and a specimen was deposited in the Botanical Survey of India, Tamilnadu Agricultural University Campus (Coimbatore, India). All the other chemicals used were of analytical or reagent grade.

### 2.2. Extraction and Isolation of Mucilage

The fruits were thoroughly washed with water. A fruit pulp was made and initially heated with steam at 80 °C for 3 min to inhibit enzymatic browning reactions. The fruit pulp was homogenized with three times its weight of water. The solution was soaked for 19–20 h for the release of mucilage into the water. Then the solution was squeezed and filtered using a muslin cloth-bag. The filtrate was collected, and the mucilage was precipitated with three times of its volume of ethanol. Thus, cream-coloured precipitate was obtained and washed with acetone three times (10 mL each). The obtained solid was dried initially under vacuum for 17–18 h followed by sunlight exposure for 30 min to yield 5.5 g mucilage/kg of fruits. Finally, the isolated mucilage was powdered, passed through the sieve number 60, and stored inside the desiccator for the future experiments or the subsequent tests.

### 2.3. Physicochemical and Characterization of the Isolated Mucilage

The physicochemical properties such as identification tests, organoleptic properties, solubility, swelling index, loss on drying, viscosity, cytotoxicity, X-ray diffraction (XRD), scanning electron microscopy (SEM), compatibility studies of thermogravimetric analysis (TGA), differential thermal analysis (DTA), differential scanning calorimetry (DSC) and Fourier-transform infrared spectroscopy (FTIR), flow properties, pH and microbial load of the mucilage were determined according to the corresponding recommended protocols [16,17,18].

### 2.4. Formulations of Paracetamol Granules and Their Key Compositions

The mucilage was evaluated for its binding and disintegrating properties in tablets of paracetamol (a model drug). Binding properties of granules were evaluated for each of the three different formulations containing varying concentrations (3%, 6%, and 9%) of *Coccinia grandis* mucilage as test binder, starch, and PVP, respectively [19] by a wet granulation technique (Table 1). Disintegrating properties of granules were evaluated for each of the two different formulations containing varying concentrations (2%, 3%) of mucilage (test disintegrant), and MCCP, CCS, and SSG (standard disintegrants) by a wet granulation technique (Table 2).

### 2.5. Evaluation of Granules

The flow properties of the granules were determined for their bulk density, tapped density, (compressibility index), Carr’s index, Hausner’s ratio, and angle of repose as per the reported protocols [20].

### 2.6. Production and Evaluation of Tablets Formulations

The different batches of granules were formulated and compressed into an average weight of 400 mg per tablet using a rotary tablet compression machine (Shakti Pharmatech Pvt Ltd., Chennai, India) fitted with a concave punch and die set. The prepared tablets were evaluated for their hardness, friability, weight variation, assay, disintegration time, in vitro dissolution profiles, and accelerated stability studies using the suitable methods specified in the Indian Pharmacopoeia (IP) 2007 [21]. 

## 3. Results and Discussion

### 3.1. Physicochemical Characterization of Isolated Mucilage Powder

The physicochemical and microbiological properties of the mucilage were determined and the results are collected in Table 3 and Table 4. The reports of the identification tests of mucilage indicated the presence of carbohydrate, mucilage and polysaccharides through the Molisch’s, ruthenium and iodine tests, respectively. The extracted and purified mucilage was evaluated for its viscosity, bacterial load, and pH. The microbial count of bacteria and fungi was found to be less than 300 and 100 CFU (colony forming units) per gram of mucilage, respectively. The pH of the mucilage was found to be 6.7. Since the pH value of this mucilage is near to neutral, it may be less irritating on the gastrointestinal tract (GIT) and hence, it will be suitable for the uncoated tablets formulations. The flow- properties of the mucilage powders were determined by Carr’s index, Hausner’s ratio and angle of repose and were found to be >23, >1.25, and 36°–40°, respectively, all of which indicated acceptable flow properties. 

### 3.2. Thermal Methods of Analysis

The drug-excipient compatibility studies were analyzed using thermal methods of analysis such as TGA, DTA, and DSC. The results of thermal analyses of mucilage of *C. grandis* demonstrated that there is no change in melting point which confirmed that there is neither changes in the colour of the drug nor had any interaction. 

### 3.3. Thermogravimetric Analysis (TGA)

**Figure 1:**
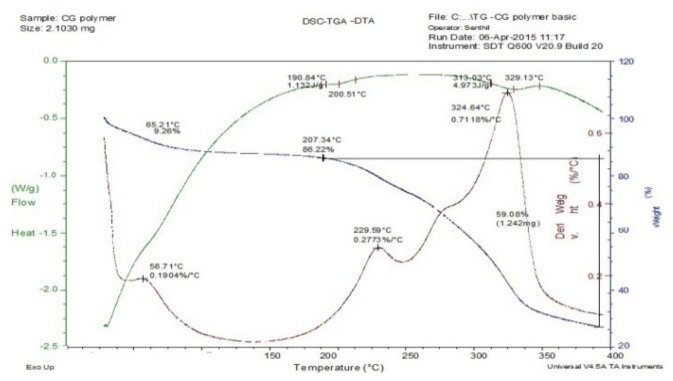
DSC, TGA, DTA of mucilage (CGM).

**Figure 2:**
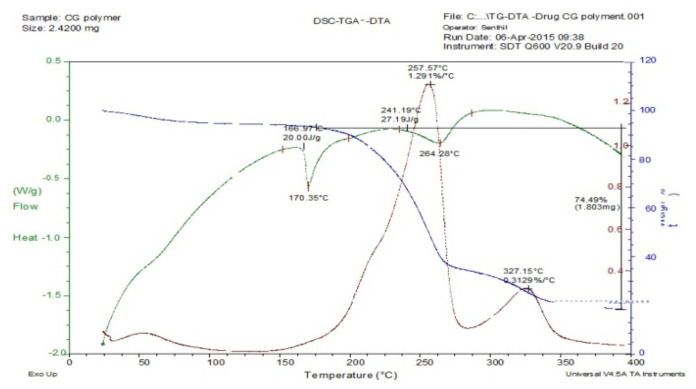
DSC, TGA, DTA of paracetamol + CGM.

The thermogravimetric curve of the *Coccinia grandis* mucilage (CGM) is presented in Figure 1. It clearly shows the weight loss corresponding to the loss of water around 25–190 °C. The mucilage underwent 9.26% weight loss at 65.21 °C which implied that CGM had good thermal stability. The curve also indicated that the mucilage did not decompose before 200 °C and starts to decompose at 207.34 °C. Hence, water is formed by intra- and inter-molecular condensation of the mucilage hydroxyls are the main products of decomposition at a temperature below 450 °C. The TGA of CGM and drug mixture showed no major interaction of CGM and drug (Figure 2), hence the mixtures are compatible with each other.

### 3.4. Differential Scanning Colorimetry (DSC)

The DSC curve of CGM (Figure 1) demonstrated that it undergoes a glass transition at a temperature to 190.84 °C (1.132 J/G). The continuous (broad) endothermic transition that recedes the glass transition is an indicative of moisture loss in the sample and start decomposing at 313.03 °C (4.973 J/G). The sample DSC overlap curve of CGM, drug, and its mixture (Figure 2) showed no additional peaks. Hence, it can be concluded that there is no physical interaction occurred in the mixture of CGM with the drug paracetamol.

### 3.5. Differential Thermal Analysis (DTA)

**Figure 3:**
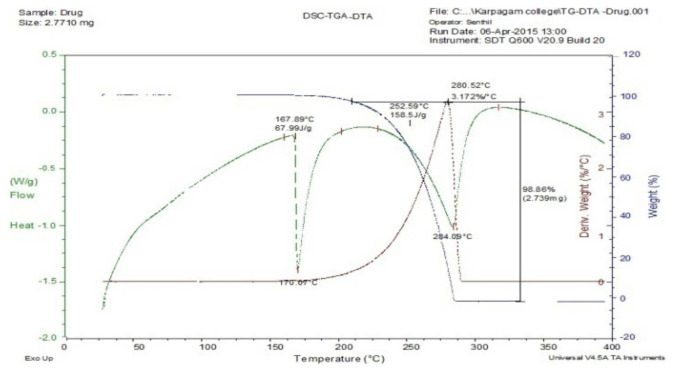
DSC, TGA, DTA of Paracetamol.

The DTA curve of mucilage undergoes crystallization at a temperature of 56.71 °C (0.1904%/°C). The mucilage started to melt at 229.59 °C (0.2773%/°C) based on the analysis conducted using DTA as shown in Figure 1. The DTA of the mixture of paracetamol drug and mucilage displayed no major interaction between CGM and drug (Figure 2). Hence, the mucilage and drug were compatible with each other. Figure 3 presents DSC, TGA, DTA of the model drug paracetamol alone.

### 3.6. FTIR Analysis

**Figure 4:**
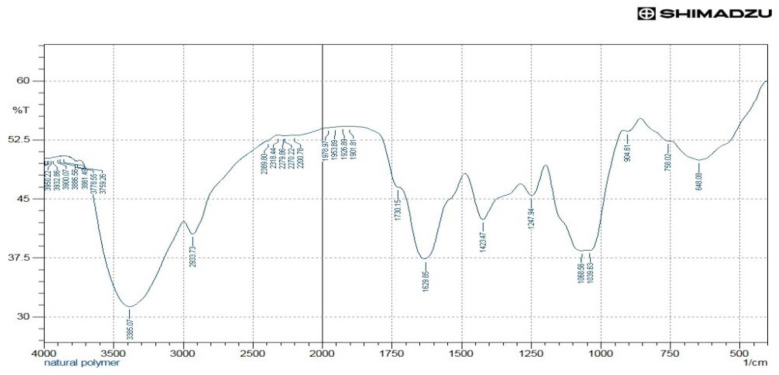
FTIR analysis of the mucilage.

**Figure 5:**
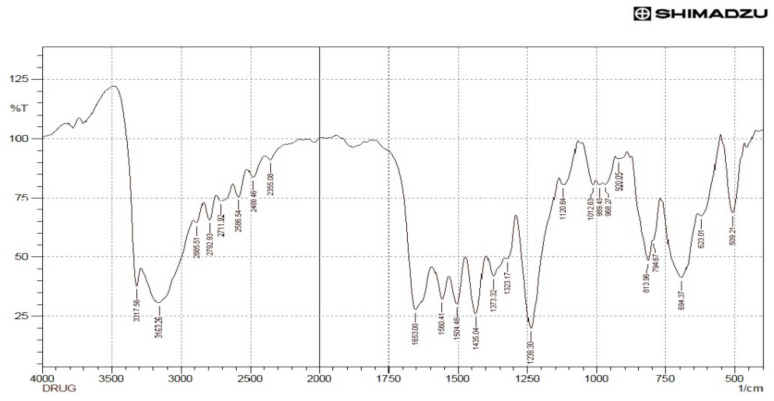
FTIR analysis of paracetamol.

**Figure 6:**
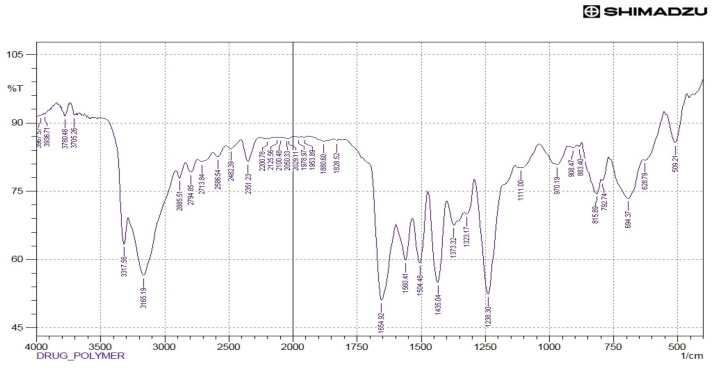
FTIR analysis of the mucilage (CGM) + paracetamol.

The drug-excipient interaction was also verified by comparing the FTIR spectrum of the physical mixture of the drug with the mucilage of *C. grandis* with FTIR spectrum of the pure drug paracetamol. The main components of the mucilage (CGM) may be galactose, rhamnose, and galacturonic acid as shown in the FTIR spectrum (Figure 4). A broad stretching peak observed at 3385.07 cm^−1^ indicated the presence of the O-H functional group as the main functional group of carbohydrates/polysaccharides as the three main components of CGM. The O-H groups can bind with water molecules and produce bound moisture to the mucilage components. The existence of O-H groups represents the hydrophilicity of the mucilage. Besides, O-H group, the carbonyl (C=O) stretching peak observed at 1629.85 cm^−1^ in the FTIR confirmed the chemical nature of CGM containing mostly carbohydrate molecules, which is considered to be the main backbone of the mucilage. The FTIR spectrum of paracetamol is presented in the Figure 5. The FTIR spectrum of the mixture of mucilage and drug is shown in Figure 6 that disclosed distinctive stretching vibrational peaks at 3317.56 cm^−1^, 3165.19 cm^−1^, 1654.92 cm^−1^, and 1560.41 cm^−1^ corresponding to the functional groups present in the mucilage and the drug. Further, there are no abnormal peaks, and the functional groups of the drug are seen intact which in turn demonstrated that the drug is compatible with mucilage.

The vibrational frequencies of various functional groups of the pure drug remained intact in the physical mixture containing *C. grandis* according to the Figure 6. Thus, it was concluded that there was no major interaction occurred between the drug and *C. grandis* used in the current study. Hence, the mucilage can be used effectively as a pharmaceutical excipient in the tablet formulations.

### 3.7. X-ray Powder Diffraction Study

**Figure 7:**
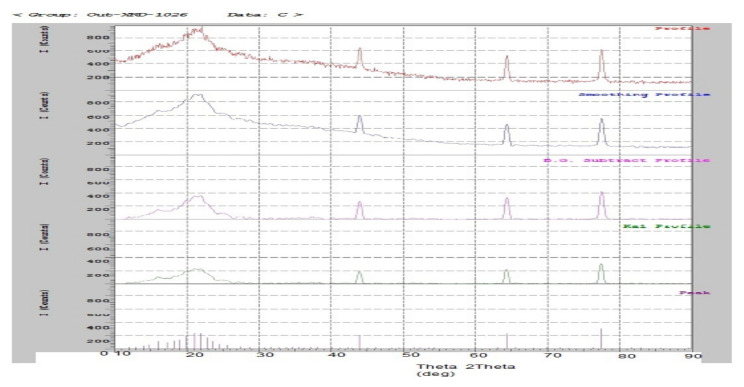
XRD analysis of mucilage (CGM).

The surface morphology of the mucilage powder was observed by the X-ray diffraction (XRD) method and the results are shown in the Figure 7. From the spectra obtained through XRD, it was deduced that the mucilage powder showed the presence of numerous halos with weak peaks which in turn confirmed the amorphous nature of the material. 

### 3.8. Scanning Electron Microscopy

**Figure 8:**
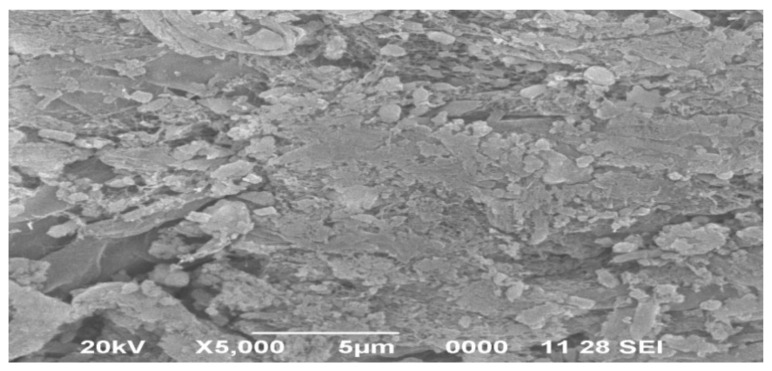
SEM analysis of mucilage in ×5000.

The surface morphology of the mucilage was also observed under scanning electron microscope (SEM) analysis and the result at ×5000 is shown in Figure 8. Further, the images of SEM results under different magnification are presented in Supplementary Materials (Figures S1–S3). The images of the mucilage revealed that the surface of particles is mostly seen as aggregates of rough, irregular in size and shapes and dimensions which were fibrous in nature, subsequently confirmed the amorphous nature of the material. 

### 3.9. In Vitro Cytotoxicity Evaluation

The toxicity study of the mucilage was performed in the human embryonic kidney (HEK) cell line. The cells were maintained at 37 °C, 5% CO_2_, 95% air, and 100% relative humidity. The concentration Vs absorbance and percentages of cell viability of test sample were calculated with control sample and are collected in Table 5 and Table 6 and the images are presented in the Supplementary Materials (Figures S4–S10). The HEK cell line had no morphological changes, and the cell viability was nearly 100% (i.e., above 80%). The reduction of MTT by cells indicated the mitochondrial activity, which may be interpreted as a proof of cell viability. It was concluded that the CGM unable to induce cytotoxic effects to the normal cells at the used concentrations. 

### 3.10. Evaluation of the Formulated Granules for Flow Properties

The flow properties of the prepared granules of different batches were determined and the results are shown in Table 7 and Table 8. A decrease in flowability was observed when the concentration (as a binding agent) of the mucilage is increased. While compared with standard starch and PVP granules, the flow property of the formulated granules differs slightly. If the concentration of the mucilage is increased as a as a disintegrating agent, then the flowability is also increased, correspondingly. This value slightly deviates from the flow properties of the granules prepared from standard excipients (MCCP, CCS, and SSG). The Carr’s index, Hausner’s ratio and angle of repose values of the granules prepared using the mucilage were found to be <23, <1.25, and 25°–30°, respectively. Hence, it was concluded that all the granules exhibited excellent flow properties.

### 3.11. Evaluation of Tablets Using Isolated Mucilage as a Binding Agent

By employing the isolated mucilage as a binding agent at three different percentages, a few batches of tablets were manufactured. For comparison purposes, starch and PVP were used as standard binding agents in the evaluation. The formulated tablets were evaluated and the results of various parameters such as weight variation, hardness, thickness, diameter, friability, disintegration time, and assay were shown in the Table 9. All the prepared batches of tablets exhibited a good content uniformity. The hardness of the tablets proportionally increasing with an increase in the percentage of used binding agents. Tablets prepared with 9% mucilage showed more hardness when compared to the tablets prepared using 3% or 6%. The friability test values were found to be lower with an increase in the concentration of the binder, however, the overall friability falls within the specified IP limits. The disintegration time of the tablets increases as increase in the concentration of the binder from 3% to 9%. This behaviour can be attributed to the swelling properties of the mucilage. But the overall disintegration time values were within the IP prescribed limits.

### 3.12. Evaluation of Tablets Using Isolated Mucilage as a Disintegrating Agent

Different batches of tablets were prepared using isolated mucilage as a suitable disintegrating agent at two different concentrations. For comparison, MCCP, CCS, and SSG were employed as standard disintegrating agents. The prepared tablets were evaluated and the results of their weight variation, hardness, thickness, diameter, friability, disintegration time, and assay are presented in Table 10. All the batches of tablets exhibited a good content uniformity, and a hardness between 4.0 to 4.5 kg/cm^2^ was observed. The friability of the tablets was found to be within the approved range of less than 0.5 to 1% as per IP. The disintegration time of the isolated mucilage was determined to be within 15 min as per IP limits which indicated a slight difference or almost equal to the standard disintegrants. The disintegration time of tablets was found to be decreased with an increase in the concentration of the used mucilage. When the mucilage concentration was increased above 3%, the disintegration time also increased, accordingly. Thus, the mucilage acts as a suitable disintegrating agent, at an ideal concentration of less than 3%. 

### 3.13. In Vitro Dissolution Studies of Tablets Using Isolated Mucilage as a Binding Agent

**Figure 9:**
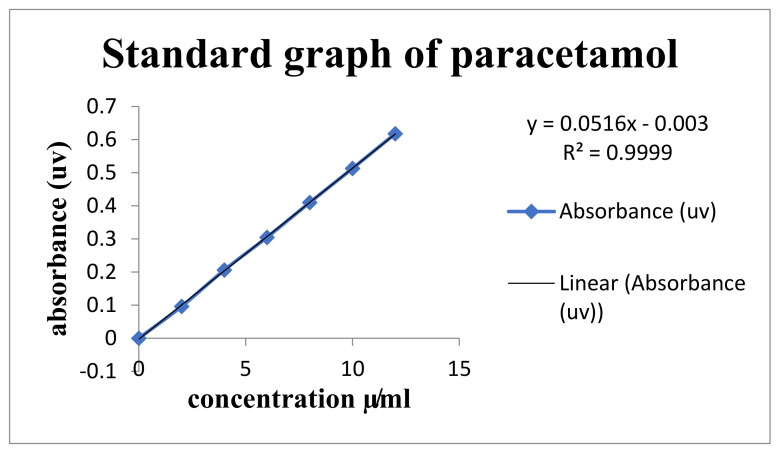
Standard graph of paracetamol drug.

**Figure 10:**
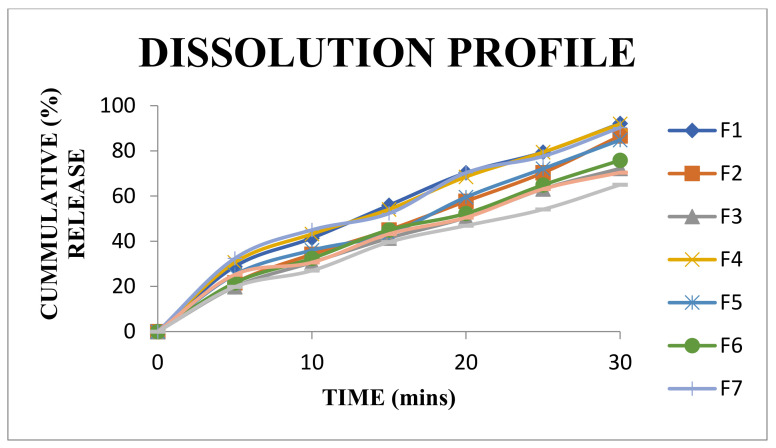
Comparative dissolution profiles for formulations F1 to F9.

In vitro dissolution profile of tablets is shown in Figure 9 and Figure 10, Table 11 and Table 12. The results of this study showed that the drug release from the tablets prepared using the mucilage with 3% and 6% concentrations were found to be more than 80%, whereas using 9% concentration of the mucilage, the drug release of 80% occurred in 30 min. The drug release was found to be increased with a decrease in the concentration of the mucilage. From the Figure 10, the drug release of F1 and F2 batches showed a sharp increase, whereas F3 showed minimal drug release while comparing with other standard batches. The friability and disintegration time of all the formulations were identified to be within the IP standards. The drug release of F1 and F2 formulations is within IP standard, except the formulation F3. 

### 3.14. In Vitro Dissolution Studies of Tablets Using Isolated Mucilage as a Disintegrating Agent

**Figure 11:**
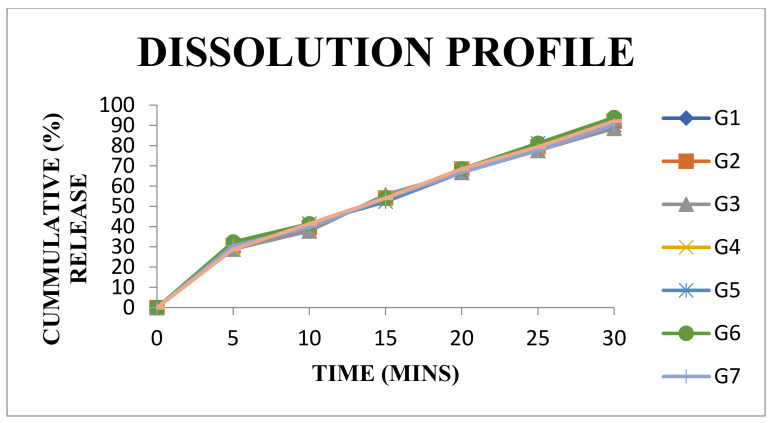
Comparative dissolution profiles for formulations G1 to G8.

In vitro dissolution profiles of tablets are shown in Figure 9, Figure 10 and Figure 11 and the parameters are presented in the Supplementary Materials as Tables S1–S3. The drug release from the tablets prepared using the mucilage with 2% and 3% concentrations were found to be more than 80% in 30 min (as per IP limits). The drug release was found to be increased with an increase in the concentration of mucilage (as a disintegrant). The formulation of G1 (2%) and G2 (3%) showed good disintegrant and the drug release was accomplished as above 80% (within the limit as per IP). Above 3% of the mucilage concentration, the disintegration time was slightly increased and conversely, the dissolution time decreased. Hence, the mucilage acts as an appropriate disintegrating agent within the concentration range of 1 to 3%.

### 3.15. Statistical Analysis

Statistical analysis of in vitro dissolution of the mucilage (CGM) with starch and PVP (standard binding agents) was performed using the DD SOLVER software for difference factor (f1), similarity factor (f2), and Rescigno index (ξ) values. The results showed that the comparison of mean (R) reference and mean (T) test values of difference factor (f1) was below 15, similarity factor (f2) was above 50 and the Rescigno index was almost 0 (Table 11). These values displayed the binding properties of the isolated mucilage are similar to that of starch and PVP. 

Statistical analysis of in vitro dissolution profile comparison of the mucilage (CGM) with MCCP, CCS, and SSG (standard disintegrating agents) was conducted using DD SOLVER software for difference factor (f1), similarity factor (f2), and Rescigno index (ξ) values. The results demonstrated that the comparison of mean (R) reference and mean (T) test values of difference factor (f1) was below 15, similarity factor (f2) was above 50, and the Rescigno index was almost 0 (Table 12). These values presented the disintegrating properties of isolated mucilage which are like that of standard disintegrating agents (MCCP, CCS, and SSG).

### 3.16. Accelerated Stability Study

The stability studies at 25 °C/60% RH, 30 °C/65% RH, and 40 °C/75% RH, respectively were maintained for 1, 30, and 90 days as per ICH guidelines. The tablet formulation (F2) was carried out and the results are presented in Table 13. The hardness of tablets was increased slightly, but there were no significant changes in their physical appearance. The drug disintegration time and drug contents of the tablets were analyzed after 1, 30, and 90 days of the storage and there were no significant changes in the disintegration time, and drug content, correspondingly. This indicated that there were no significant changes in the physical as well as chemical characteristics of the formulations. Hence, it can be concluded that the developed formulation was stable and retained the crucial pharmaceutical properties over a period of three months.

## 4. Conclusions

It can be concluded that the mucilage isolated from *Coccinia grandis* showed the presence of constituents such as carbohydrates, and polysaccharides. It has a near neutral pH, which indicated that this mucilage does not irritate the GIT and is suitable for formulating an uncoated tablet. The compatibility studies through thermal methods of analysis and FTIR showed that there were no major interactions occurred between the mucilage and the model drug (paracetamol). The in vitro toxicity study revealed that this mucilage can be employed safely as a pharmaceutical excipient. The flow properties of granules prepared with mucilage had as good compressibility as that of the granules formulated using starch and PVP. Post-compression parameter analysis suggested that tablets formulated with mucilage had better hardness and friability than the tablets prepared with starch and PVP. As the binding concentration of mucilage is increased, the disintegration time also increased, like that of the tablets prepared with starch and PVP. The formulations exhibited a better and more consistent release as compared to the standard formulations using starch and PVP (standard binders). Considering all the above parameters, our study evidenced a good potential of the mucilage as a binder for tablet formulations. The disintegrant properties of the mucilage was studied in comparison with the standard super disintegrants (MCCP, CCS, and SSG.) The mucilage concentration at 3% and below 3% acted as a super disintegrating agent and exhibited faster tablet disintegration and drug dissolution, thereby helping for an effective therapy and improved patient compliance. Thus, the natural super disintegrant, the mucilage can be effectively used as a suitable disintegrant in formulating solid dosage form of tablets. Further, this work can be extended to in vivo toxicity assessment for its safety confirmation, besides predicting its sustaining action. 

## Figures and Tables

**Table 1:** Composition of paracetamol tablet formulation using the different binding agents of *Coccinia Grandis* mucilage, Starch and PVP.

Ingredients	F1 (3%)	F2 (6%)	F3 (9%)	F4 (3%)	F5 (6%)	F6 (9%)	F7 (3%)	F8 (6%)	F9 (9%)
Paracetamol	250	250	250	250	250	250	250	250	250
Starch	125	113	101	125	113	101	125	113	101
mucilage (Binder)	12	24	36	-	-	-	-	-	-
Starch (Binder)	-	-	-	12	24	36	-	-	-
Polyvinylpyrrolidone (Binder)	-	-	-	-	-	-	12	24	36
Sodium methylparaben	0.8	0.8	0.8	0.8	0.8	0.8	0.8	0.8	0.8
Sodium propylparaben	0.4	0.4	0.4	0.4	0.4	0.4	0.4	0.4	0.4
Demineralised water	q.s	q.s	q.s	q.s	q.s	q.s	q.s	q.s	q.s
Talc	8	8	8	8	8	8	8	8	8
Magnesium stearate	4	4	4	4	4	4	4	4	4
Total weight	400	400	400	400	400	400	400	400	400

Note: All the above ingredients quantities are mg/tablet.

**Table 2:** Composition of paracetamol tablet formulation using the different disintegrating agents of *Coccinia grandis* mucilage, MCCP, CCS, and SSG.

Ingredients	G1 (2%)	G2 (3%)	G3 (2%)	G4 (3%)	G5 (2%)	G6 (3%)	G7 (2%)	G8 (3%)
Paracetamol	250	250	250	250	250	250	250	250
Starch (diluent)	99	95	99	95	99	95	99	95
Starch (Binder)	30	30	30	30	30	30	30	30
mucilage	8	12	-	-	-	-	-	-
Microcrystalline cellulose powder	-	-	8	12	-	-	-	-
Croscarmellose sodium	-	-	-	-	8	12	-	-
Sodium starch glycollate	-	-	-	-	-	-	8	12
Sodium methylparaben	0.8	0.8	0.8	0.8	0.8	0.8	0.8	0.8
Sodium propylparaben	0.4	0.4	0.4	0.4	0.4	0.4	0.4	0.4
Demineralised water	q.s	q.s	q.s	q.s	q.s	q.s	q.s	q.s
Talc	8	8	8	8	8	8	8	8
Magnesium stearate	4	4	4	4	4	4	4	4
Total weight	400	400	400	400	400	400	400	400

Note: All the above ingredients quantities are mg/tablet.

**Table 3:** Identification test results of the mucilage.

Tests	Observed	Results
Molisch’s test:	Violet green colour present at junction of two layers	Carbohydrate present
Ruthenium test:	Pink colour developed	Mucilage present
Iodine test:	No colour present in solution	Polysaccharides present

**Table 4:** Results of Physicochemical characterization of *Coccinia grandis* mucilage.

Parameters	Observed
Organoleptic properties	White colour, amorphous nature, tasteless, characteristic odour.
Solubility	Slightly soluble in hot water, in cold water forming viscous colloidal solution and practically insoluble in acetone, ethanol, chloroform and other organic solvents.
Loss on drying (%)	5.7%
Swelling index in distilled water	46.3%
Bulk density	0.083 g/cm^3^
Tapped density	0.125 g/cm^3^
Carr’s index	33.6
Hausner’s ratio	1.50
Angle of repose (°)	39.5°
pH (1% *w*/*v*)	6.7
Total Ash (%)	3.0%
Water-soluble ash (%)	6.7%
Acid insoluble ash (%)	0.5%
Viscosity (1% *w*/*v* solution)	4.9 cps
Total Microbial (Load) count Bacteria: (CFU/g) Fungi: CFU/g)	107 64

**Table 5:** Concentration vs. absorbance of cell viability of test and control.

S. No	Concentration (µg/mL)	Absorbance
1	12.5	0.451
2	25	0.446
3	50	0.413
4	100	0.388
5	200	0.367

Average control absorbance = 0.451.

**Table 6:** Concentrations vs. % cell viability.

S. No	Concentration (µg/mL)	% Cell Viability
1	12.5	99.92
2	25	98.81
3	50	91.65
4	100	85.96
5	200	81.38

**Table 7:** Flow properties of formulated granules (Binding agents).

Binders	CGM			STARCH			PVP		
Formulations Code	F1 (3%)	F2 (6%)	F3 (9%)	F4 (3%)	F5 (6%)	F6 (9%)	F7 (3%)	F8 (6%)	F9 (9%)
Parameters									
Bulk density (g/mL)	0.438 ± 0.00	0.446 ± 0.00	0.446 ± 0.00	0.434 ± 0.00	0.442 ± 0.00	0.446 ± 0.00	0.438 ± 0.00	0.446 ± 0.00	0.442 ± 0.00
Tapped density (g/mL)	0.510 ± 0.00	0.500 ± 0.00	0.495 ± 0.00	0.526 ± 0.00	0.500 ± 0.00	0.490 ± 0.00	0.505 ± 0.00	0.490 ± 0.00	0.480 ± 0.00
Carr’s index (%)	14.1 ± 0.00	10.8 ± 0.00	9.9 ± 0.01	17.5 ± 0.01	11.6 ± 0.00	9.0 ± 0.03	13.3 ± 0.04	9.0 ± 0.03	7.9 ± 0.00
Hausner’s ratio	1.16 ± 0.00	1.12 ± 0.00	1.11 ± 0.01	1.21 ± 0.00	1.13 ± 0.00	1.10 ± 0.01	1.15 ± 0.00	1.10 ± 0.01	1.10 ± 0.02
Angle of repose (°)	29.3°	25.1°	26.2°	29.7°	26.4°	25.9°	29.9°	28.4°	27.8°

CGM = mucilage, PVP = polyvinylpyrrolidone.

**Table 8:** Flow properties of formulated granules (Disintegrating agents).

Disintegrants	CGM		MCCP		CCS		SSG	
Formulations Code	G1 (2%)	G2 (3%)	G3 (2%)	G4 (3%)	G5 (2%)	G6 (3%)	G7 (2%)	G8 (3%)
Parameters								
Bulk density (g/mL)	0.446 ± 0.00	0.442 ± 0.00	0.446 ± 0.00	0.442 ± 0.00	0.442 ± 0.00	0.446 ± 0.00	0.442 ± 0.00	0.446 ± 0.00
Tapped density (g/mL)	0.495 ± 0.00	0.500 ± 0.00	0.500 ± 0.00	0.505 ± 0.00	0.500 ± 0.00	0.495 ± 0.00	0.505 ± 0.00	0.490 ± 0.00
Carr’s index (%)	9.9 ± 0.01	11.6 ± 0.00	10.8 ± 0.01	12.5 ± 0.03	11.6 ± 0.00	9.9 ± 0.01	12.5 ± 0.03	9.0 ± 0.03
Hausner’s ratio	1.11 ± 0.01	1.13 ± 0.01	1.12 ± 0.01	1.14 ± 0.00	1.13 ± 0.01	1.11 ± 0.01	1.14 ± 0.00	1.10 ± 0.01
Angle of repose (°)	28.3°	29.1°	28.7°	29.7°	28.4°	28.9°	29.2°	28.8°

CGM = mucilage, MCCP = microcrystalline cellulose powder, CCS = croscarmellose sodium, SSG = sodium starch glycollate.

**Table 9:** Evaluation of tablets using different binding agents.

Binders	CGM			STARCH			PVP		
Formulations Code	F1 (3%)	F2 (6%)	F3 (9%)	F4 (3%)	F5 (6%)	F6 (9%)	F7 (3%)	F8 (6%)	F9 (9%)
Parameters									
Weight variation (mg)	400.1	400.0	401.4	400.0	401.1	400.2	401.0	401.2	400.1
Hardness (kg/cm^2^)	4.0	4.5	5.5	4.0	4.5	5.0	4.5	5.0	6.5
Thickness (mm)	4.8	4.8	5.0	4.8	5.0	4.8	4.9	5.0	4.8
Diameter (mm)	10.14	10.14	10.12	10.14	10.12	10.14	10.14	10.14	10.14
Friability (% *w*/*w*)	0.97	0.68	0.49	0.85	0.61	0.47	0.77	0.52	0.41
Disintegration time (min)	2 min/5 s	4 min/2 s	6 min/28 s	1 min/48 s	3 min/52 s	5 min/22 s	1 min/54 s	5 min/49 s	13 min/36 s
Assay (%)	99.7	99.6	98.9	100.1	98.8	99.8	98.7	100.2	99.9

**Table 10:** Evaluation of tablets using different disintegrating agents.

Disintegrants	CGM		MCCP		CCS		SSG	
Formulations Code	G1 (2%)	G2 (3%)	G3 (2%)	G4 (3%)	G5 (2%)	G6 (3%)	G7 (2%)	G8 (3%)
Parameters								
Weight variation (mg)	400.0	400.3	400.1	401.0	400.1	399.9	401.2	400.7
Hardness (kg/cm^2^)	4.0	4.0	4.0	4.5	4.0	4.0	4.5	4.0
Thickness (mm)	4.8	4.8	4.8	4.9	4.8	4.8	5.0	4.9
Diameter (mm)	10.14	10.14	10.14	10.12	10.14	10.14	10.14	10.14
Friability (% *w*/*w*)	0.44	0.42	0.45	0.42	0.48	0.43	0.44	0.47
Disintegration time (min)	2 min/58 s	2 min/22 s	2 min/51 s	2 min/36 s	2 min/49 s	2 min/12 s	2 min/51 s	2 min/17 s
Assay (%)	98.9	99.9	99.7	100.1	99.8	98.9	99.6	98.7

**Table 11:** Statistical factors of CGM compared with STARCH and PVP as binding agents.

Binders	CGM vs. STARCH			CGM vs. PVP		
Formulations Code	F1 vs. F4 (3%)	F2 vs. F5 (6%)	F3 vs. F6 (9%)	F1 vs. F7 (3%)	F2 vs. F8 (6%)	F3 vs. F9 (9%)
Statistical Factors						
Difference factor (f1)	2.01	3.90	4.93	3.91	13.99	9.98
Similarity factor (f2)	87.48	80.91	78.15	76.69	54.37	63.82
Rescigno index (ξ)	0.0113	0.0207	0.0252	0.0209	0.0606	0.0468

**Table 12:** Statistical factors of CGM compared with MCCP, CCS and SSG as disintegrating agents.

Disintegrants	CGM vs. MCCP		CGM vs. CCS		CGM vs. SSG	
Formulations Code	G1 vs. G3 (2%)	G2 vs. G4 (3%)	G1 vs. G5 (2%)	G2 vs. G6 (3%)	G1 vs. G7 (2%)	G2 vs. G8 (3%)
Statistical Factors						
Difference factor (f1)	2.00	1.49	1.99	1.94	1.03	0.99
Similarity factor (f2)	87.71	89.75	87.51	87.51	92.04	92.04
Rescigno index (ξ)	0.0099	0.0070	0.0114	0.0098	0.0059	0.0057

**Table 13:** Accelerated stability studies of F2.

S. No	Temperature	Time in Days	Physical Change	Hardness kg/cm^2^	Disintegration Time (min)	Drug Content (%)
1.	25 °C	1	No change	4.5	4 min/22 s	99.4
		30	No change	5.0	5 min/14 s	98.9
		90	No change	5.0	5 min/27 s	98.3
2.	30 °C	1	No change	4.5	4 min/15 s	99.6
		30	No change	5.0	5 min/32 s	98.7
		90	No change	5.5	6 min/40 s	98.1
3.	40 °C	1	No change	4.5	4 min/5 s	98.9
		30	No change	5.0	5 min/34 s	98.2
		90	No change	5.0	5 min/48 s	97.8

## Data Availability

Not applicable.

## Supplementary Materials

The following supporting information can be downloaded at: https://www.mdpi.com/article/10.3390/polym14010215/s1, Figure S1: SEM analysis of mucilage in ×20,000; Figure S2: SEM analysis of mucilage in ×55,000; Figure S3: SEM analysis of mucilage in ×30,000; Figure S4: Graph of concentration vs. % cell viability; Figure S5: Image of cytotoxicity in 12.5 µg/mL; Figure S6: Image of cytotoxicity in 25 µg/mL; Figure S7: Image of cytotoxicity in 50 µg/mL.; Figure S8: Image of cytotoxicity in 100 µg/mL; Figure S9: Image of cytotoxicity in 200 µg/mL; Figure S10: Image of cytotoxicity of control sample; Table S1: Standard graph of paracetamol drug; Table S2. Invitro drug release of tablets using isolated mucilage and standard binders; Table S3. In vitro drug release of tablets using isolated mucilage and standard disintegrants.
