# Supplementary material for: Mucilage of Coccinia grandis as an Efficient Natural Polymer-Based Pharmaceutical Excipient
Source: Polymers (Basel). 2022 Jan 5;14(1):215. doi: 10.3390/polym14010215 (PMC8747206; doi:10.3390/polym14010215)
Supplement: Supplementary file 1 [file polymers-14-00215-s001.zip › polymers-1530416-supplementary.pdf]

Supporting Information

Mucilage of *Coccinia grandis* as an Efficient Natural Polymer-Based Pharmaceutical Excipient †

Kumbakonam Balachandran Ilango [1,*], Senguttuvan Gowthaman [1], Kumbakonam Ilango Seramaan [2], Kumarappan Chidambaram [3,*], Mohammad F. Bayan [4], Mohamed Rahamathulla [5] and Chandrasekaran Balakumar [6]

[1] Department of Pharmaceutics, College of Pharmacy, Shree Venkateshwara College of Paramedical Sciences, Erode 638455 , Tamil Nadu, India; gowthamanpharma@gmail.com
[2] Department of Pharmaceutics, Vellalar College of Pharmacy, Erode 638012, Tamil Nadu, India; seramaan5435@gmail.com
[3] Department of Pharmacology, School of Pharmacy, King Khalid University, Abha 62529, Saudi Arabia
[4] Faculty of Pharmacy, Philadelphia University, P.O. Box 1, Amman 19392, Jordan; mbayan01@qub.ac.uk
[5] Department of Pharmaceutics, School of Pharmacy, King Khalid University, Abha 62529, Saudi Arabia; rahapharm@gmail.com
[6] Department of Pharmaceutical Chemistry, School of Pharmacy, ITM University, Gwalior 474001, Madhya Pradesh, India; dhillbalu@gmail.com
* Correspondence: kbilango04@gmail.com (K.B.I.); kumarappan@kku.edu.sa (C.K.)
† Part of this work has been submitted as a thesis by S.G. to The TN Dr MGR Medical University, India.

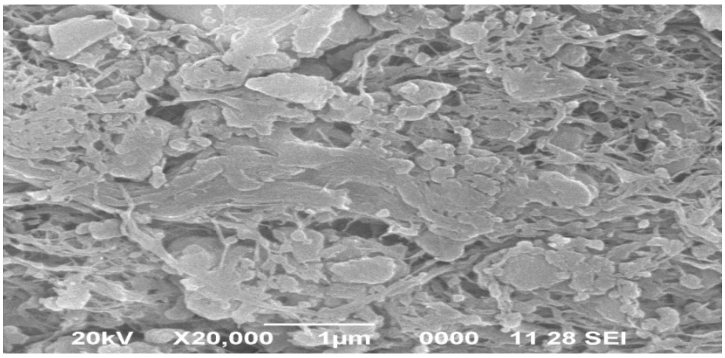

Figure S1. SEM analysis of mucilage in X20000.

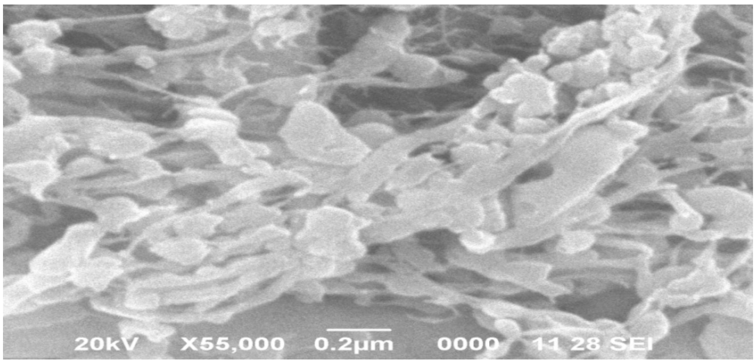

Figure S2. SEM analysis of mucilage in X55000.

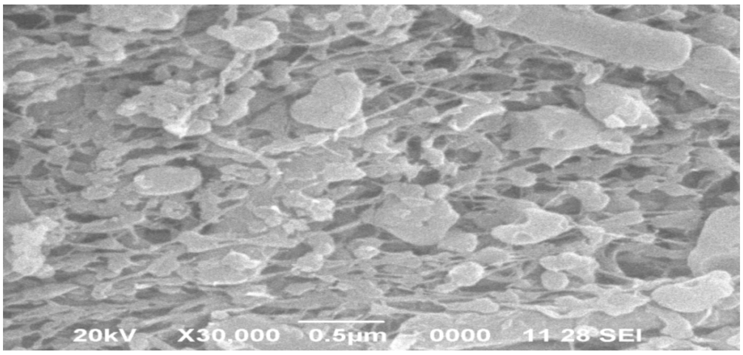

Figure S3. SEM analysis of mucilage in X30000.

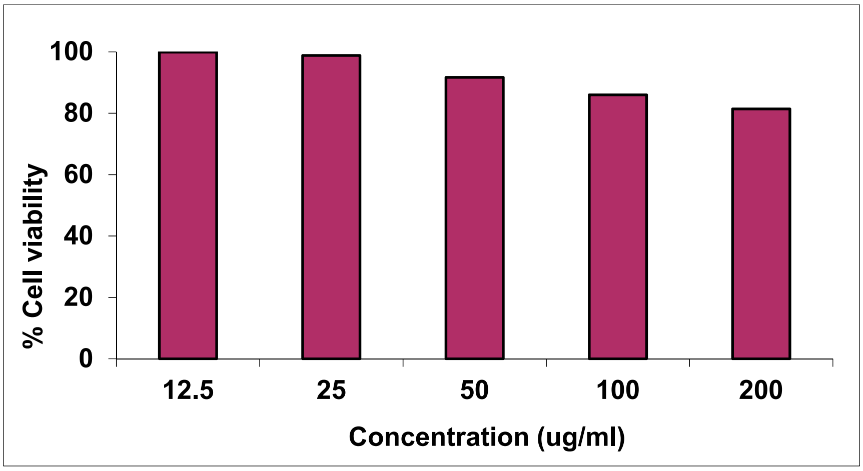

Figure S4. Graph of concentration vs % cell viability.

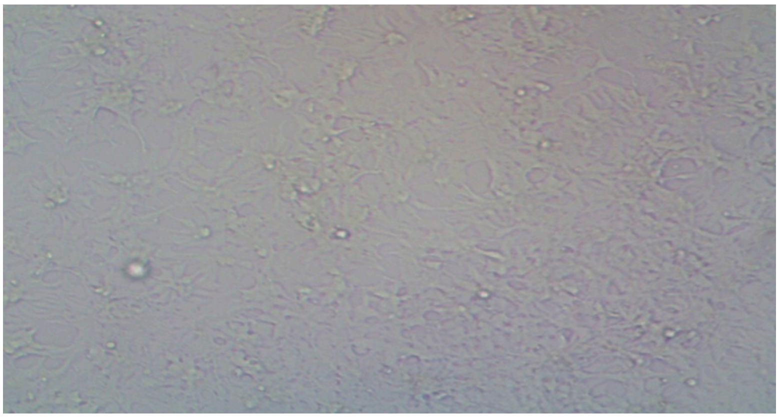

Figure S5. Image of cytotoxicity in 12.5 μg/ml.

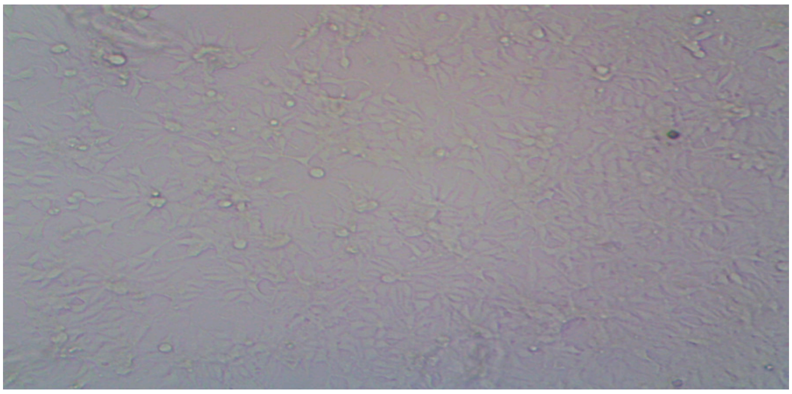

Figure S6. Image of cytotoxicity in 25 μg/ml.

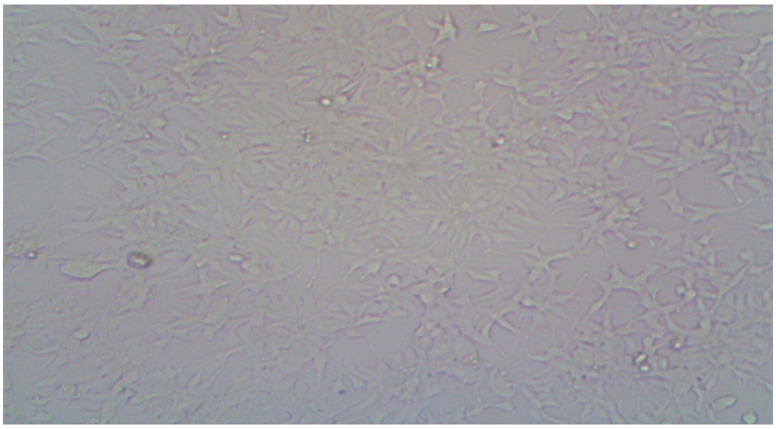

Figure S7. Image of cytotoxicity in 50 μg/ml.

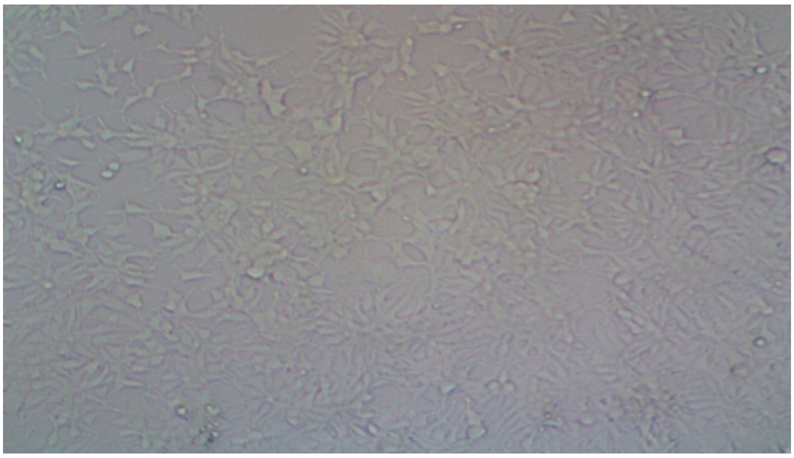

Figure S8. Image of cytotoxicity in100 μg/ml.

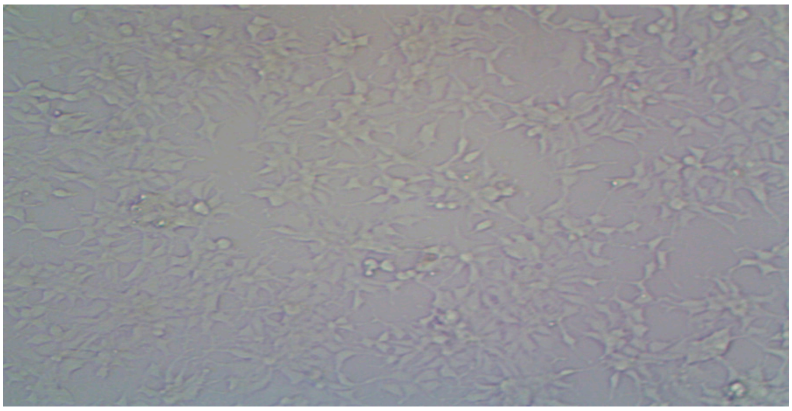

Figure S9. Image of cytotoxicity in 200 μg/ml.

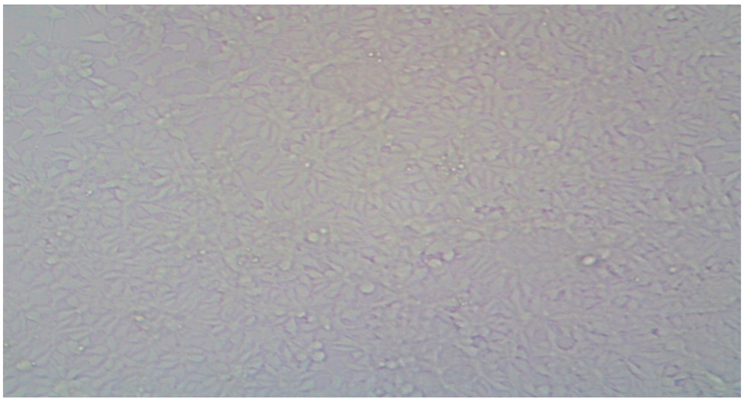

Figure S10. Image of cytotoxicity of control sample.

Table S1. Standard graph of paracetamol drug.

Concentration (µg/ml)	Absorbance (UV)
0	0
2	0.096
4	0.206
6	0.305
8	0.410
10	0.513
12	0.618

Table S2. *In vitro* drug release of tablets using isolated mucilage and standard binders.

Binders →	CGM			STARCH			PVP		
Formulations code → / Dissolution time (mins) ↓	F1 (3%)	F2 (6%)	F3 (9%)	F4 (3%)	F5 (6%)	F6 (9%)	F7 (3%)	F8 (6%)	F9 (9%)
5	28.8	21.6	19.8	30.6	25.2	21.6	32.4	25.2	19.8
10	41.4	34.2	30.6	43.2	36.0	32.4	45.0	30.6	27.0
15	55.9	45.0	41.4	54.1	43.3	45.0	52.3	43.2	39.6
20	70.3	57.7	50.5	68.5	59.5	52.3	70.3	50.5	46.9
25	79.4	70.3	63.1	79.4	72.1	64.9	77.6	63.1	54.1
30	92.1	86.6	72.2	91.9	84.8	75.8	90.3	70.4	65.0

Table S3. *In vitro* drug release of tablets using isolated mucilage and standard disintegrants.

Disintegrants →	CGM		MCCP		CCS		SSG	
Formulations code → / Dissolution time (mins) ↓	G1 (2%)	G2 (3%)	G3 (2%)	G4 (3%)	G5 (2%)	G6 (3%)	G7 (2%)	G8 (3%)
5	28.8	30.6	28.8	29.0	30.6	32.4	30.6	28.8
10	39.6	39.6	37.8	41.4	41.4	41.4	39.6	41.4
15	54.1	54.1	55.8	54.0	52.3	54.1	54.0	54.1
20	66.7	68.5	66.7	68.5	66.7	68.5	66.7	68.5
25	79.4	79.4	77.6	79.4	81.2	81.2	77.6	79.4
30	90.3	92.1	88.5	90.2	90.3	93.9	90.3	92.1
